# Dietary *Sargassum fusiforme* improves memory and reduces amyloid plaque load in an Alzheimer’s disease mouse model

**DOI:** 10.1038/s41598-019-41399-4

**Published:** 2019-03-20

**Authors:** Jeroen Bogie, Cindy Hoeks, Melissa Schepers, Assia Tiane, Ann Cuypers, Frank Leijten, Yupyn Chintapakorn, Thiti Suttiyut, Surachai Pornpakakul, Dicky Struik, Anja Kerksiek, Hong-Bing Liu, Niels Hellings, Pilar Martinez-Martinez, Johan W. Jonker, Ilse Dewachter, Eric Sijbrands, Jochen Walter, Jerome Hendriks, Albert Groen, Bart Staels, Dieter Lütjohann, Tim Vanmierlo, Monique Mulder

**Affiliations:** 1 Department of Immunology and Biochemistry, Biomedical research institute, Hasselt University, Martelarenlaan 42, 3500 Hasselt, Belgium; 2 Centre for Environmental Sciences, Hasselt University, Martelarenlaan 42, 3500 Hasselt, Belgium; 3 Department of Internal Medicine, Laboratory of Vascular Medicine, Erasmus University Medical Center, Wytemaweg 80, 3015 CN Rotterdam, The Netherlands; 4 Center of Excellence in Environment and Plant Physiology, Department of Botany, Faculty of Science, Chulalongkorn University, Bangkok, 10330 Thailand; 5 Department of Chemistry, Faculty of Science, Chulalongkorn University, Bangkok, 10330 Thailand; 6 Section of Molecular Metabolism and Nutrition, Department of Pediatrics, University of Groningen, University Medical Center Groningen, Hanzeplein 1, 9713 GZ Groningen, The Netherlands; 7 Institute for Clinical Chemistry and Clinical Pharmacology, Sigmund-Freud-Str. 25, D-53127 Bonn, Germany; 8 Key Laboratory of Marine Drugs, Ministry of Education, School of Medicine and Pharmacy, Ocean University of China, Yushan Road 5, 266003 Qingdao, China; 9 School for mental health and neuroscience, Maastricht University, Universiteitssingel 50, 6229ER Maastricht, The Netherlands; 10 Department of Neurology, Molecular Cell Biology, University of Bonn, Sigmund-Freud-Str. 25, 53127 Bonn, Germany; 11 Department of Medical Biochemistry, Academic Medical Center, University of Amsterdam, Meibergdreef 9, 1105 AZ Amsterdam, The Netherlands; 12 University of Lille - EGID, Inserm, U1011, University Hospital CHU, Institut Pasteur de Lille, F-59019 Lille, France

## Abstract

Activation of liver X receptors (LXRs) by synthetic agonists was found to improve cognition in Alzheimer’s disease (AD) mice. However, these LXR agonists induce hypertriglyceridemia and hepatic steatosis, hampering their use in the clinic. We hypothesized that phytosterols as LXR agonists enhance cognition in AD without affecting plasma and hepatic triglycerides. Phytosterols previously reported to activate LXRs were tested in a luciferase-based LXR reporter assay. Using this assay, we found that phytosterols commonly present in a Western type diet in physiological concentrations do not activate LXRs. However, a lipid extract of the 24(S)-Saringosterol-containing seaweed *Sargassum fusiforme* did potently activate LXRβ. Dietary supplementation of crude *Sargassum fusiforme* or a *Sargassum fusiforme*-derived lipid extract to AD mice significantly improved short-term memory and reduced hippocampal Aβ plaque load by 81%. Notably, none of the side effects typically induced by full synthetic LXR agonists were observed. In contrast, administration of the synthetic LXRα activator, AZ876, did not improve cognition and resulted in the accumulation of lipid droplets in the liver. Administration of *Sargassum fusiforme*-derived 24(S)-Saringosterol to cultured neurons reduced the secretion of Aβ_42_. Moreover, conditioned medium from 24(S)-Saringosterol-treated astrocytes added to microglia increased phagocytosis of Aβ. Our data show that *Sargassum fusiforme* improves cognition and alleviates AD pathology. This may be explained at least partly by 24(S)-Saringosterol-mediated LXRβ activation.

## Introduction

Alzheimer’s disease (AD) is a progressive neurological disorder characterized by an accumulation of extracellular amyloid-β (Aβ), intracellular neurofibrillary tangles, loss of synapses, neuroinflammation, and by a gradual progression of memory loss^1^. Accumulating evidence suggests a role for a disturbed cholesterol turnover in the central nervous system (CNS) in AD pathogenesis^2–11^. In line with this, stimulation of cholesterol turnover improves disease outcome in animal models of AD^2,12–18^. Liver X receptors (LXR) are master regulators of cholesterol and triglyceride turnover and suppress an inflammatory transcriptional profile via trans-repression of NFκB signaling^19^. Therefore, LXRs are promising well-studied therapeutic targets for increasing cholesterol turnover and decreasing neuroinflammation in AD^20–24^. We and others have reported that synthetic pan LXR agonists improve the cognitive phenotype in animal models of AD, decrease synaptic compensatory mechanisms, and stimulate the proteolytic degradation of Aβ by microglia^2,13,14,24–26^. However, synthetic full LXR agonists systematically cause adverse side effects, such as hypertriglyceridemia and hepatic steatosis, hampering their translation to the clinic^27–30^.

Phytosterols are structurally similar to cholesterol. However, in contrast to cholesterol, they can cross the blood-brain barrier (BBB) and accumulate in brain parenchyma^11,31–33^. Several of the more than 260 identified phytosterols, such as β-sitosterol, fucosterol, stigmasterol, schottenol, 24(S)-Saringosterol, and spinasterol, have been reported to activate LXRs *in vitro*^34–39^. Moreover, β-sitosterol and stigmasterol modulate AD pathology in *in vitro* models for AD^40–42^. Phytosterols do not induce hypertriglyceridemia and hepatic steatosis, which may be a consequence of their ABCG5/G8-mediated hepatic excretion into the bile^11,43,44^. The absence of unwanted side effects renders phytosterols interesting therapeutic candidates for inducing LXR activation in the CNS.

We aim to identify phytosterols and phytosterol-containing extracts that activate LXRs *in vitro*, to test their effect on memory performance and Aβ plaque pathology in an animal model of AD. We found that phytosterols typically present in a Western type diet or extracts from a range of Eastern plants hardly activate LXRα or LXRβ. In contrast, a lipid extract of the edible brown seaweed *Sargassum fusiforme* which contains large amounts of 24(S)-Saringosterol potently activated LXRβ. In a mouse model for AD, dietary supplementation with *Sargassum fusiforme* or its lipid extract not only increased the expression of LXR response genes in the CNS, but also improved cognition without inducing hepatic steatosis. Improved memory performance in these mice was paralleled by a strong reduction in CNS Aβ plaque load. In contrast, the selective LXRα agonist AZ876 did not counteract cognitive decline in AD mice or reduce Aβ plaque load, and induced liver steatosis. These findings indicate that *Sargassum fusiforme* is an attractive option for add-on treatments in the emerging field of nutritional neuroscience.

## Results

### Phytosterols present in a Western diet do not activate LXRs

First, we determined the capacity of phytosterols typically present in a Western diet to activate LXRs. To define cellular specificity in LXR activation *in vitro*, cells derived from peripheral tissues (HEK293.T and COS7) and the CNS (CHME3, MO3.13, N2a/APPswe) were tested. Physiologically relevant concentrations of stigmasterol, fucosterol, brassicasterol, β-sitosterol, or a mix of phytosterols did not activate LXRα or LXRβ in any of the cell lines used (Fig. S2). An increased incubation period promoted the capacity of the synthetic LXR agonist T0901317, but not of phytosterols, to activate LXRα and LXRβ (Fig. S3a,b). Although cellular uptake of phytosterols and their capacity to activate nuclear receptors improves when complexed to proteins^45,46^, pre-incubation of phytosterols with BSA did not significantly increase their ability to activate LXRα and LXRβ (Fig. S3c,d).

### An extract of *Sargassum fusiforme* activates LXRβ *in vitro*

**Figure 1:**
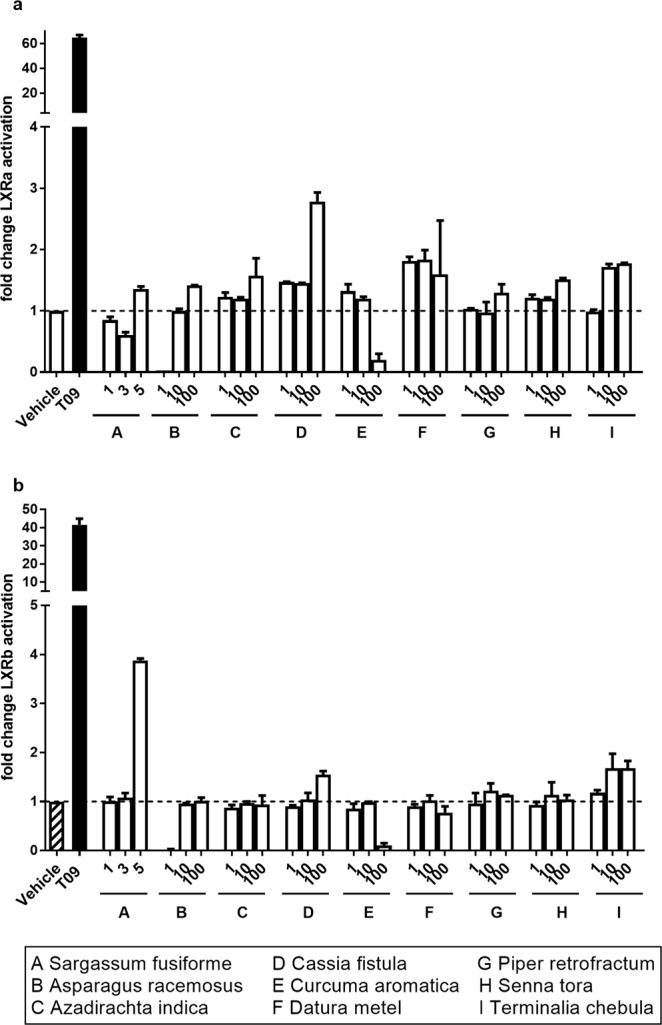
A crude lipid extract of *Sargassum fusiforme* activates LXRβ. LXRα (**a**) and LXRβ (**b**) activation was assessed with a luciferase-based reporter assay. CHME3 cells were stimulated for 18 hours with vehicle (striped bar/dotted line), 1, 3, or 5 µg/μl *Sargassum fusiforme* or 1, 10 or 100 µg/μl of the specified plant extracts. No difference was found between groups (LXRα χ^2^(7) = 11.55, p = 0.1165, LXRβ χ^2^(7) = 7.689, p = 0.3608; all datasets analysed using Kruskal-Wallis test). All results are displayed as fold change compared to vehicle control (striped bar/dotted line). Bars represent mean ± SEM (n ≥ 3).

In addition to phytosterols present in a Western diet, crude extracts of Eastern plants were screened for their capacity to activate LXRα or LXRβ. Plants were chosen based on their application in Asian traditional medicine and their presence in an Eastern diet. Extracts from *Asparagus racemosus*, *Azadirachta indica*, *Cassia fistula*, *Curcuma aromatica*, *Datura metel*, *Piper retrofractum*, *Senna tora*, and *Terminalia chebula* did not significantly activate either LXRα or LXRβ in microglial CHME3 cells (Fig. 1). However, at a dose of 5 µg/ml an extract of *Sargassum fusiforme* containing the recently identified LXRβ agonist 24(S)-Saringosterol^34^ significantly activated LXRβ but not LXRα (Fig. 1). Higher doses of *Sargassum fusiforme* induced cell death (data not shown). As *Sargassum fusiforme* showed the highest capacity to activate LXRβ, it was selected for further *in vivo* testing.

### Dietary supplementation with *Sargassum fusiforme* results in 24(S)-Saringosterol accumulation in the cerebellum and activation of LXR-response genes in AD mice

**Figure 2:**
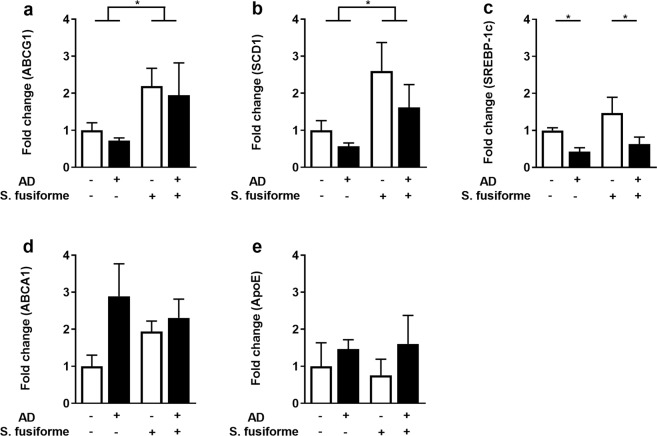
Dietary supplementation with *Sargassum fusiforme* activates LXRs in the brain. Gene expression of *Abcg1* (**a**), *Scd1* (**b**), *Srebp-1c* (**c**), *Abca1* (**d**), and *ApoE* (**e**) was measured in the brain of WT and APPswePS1ΔE9 mice (AD) fed normal chow or chow supplemented with *Sargassum fusiforme*. Two-way ANOVA revealed a diet effect for *Abcg1* (F (1, 17) = 6.244, p = 0.0230)), and *Scd1* (F (1, 15) = 7.673, p = 0.0143), and a genotype effect for *Srebp-1c* (F (1, 16) = 8.446, p = 0.0103). Gene expression was normalized to *Cyca* and *Hmbs*, and expressed as fold change compared to WT mice fed the control diet. Bars represent mean ± SEM (n ≥ 5).

**Figure 3:**
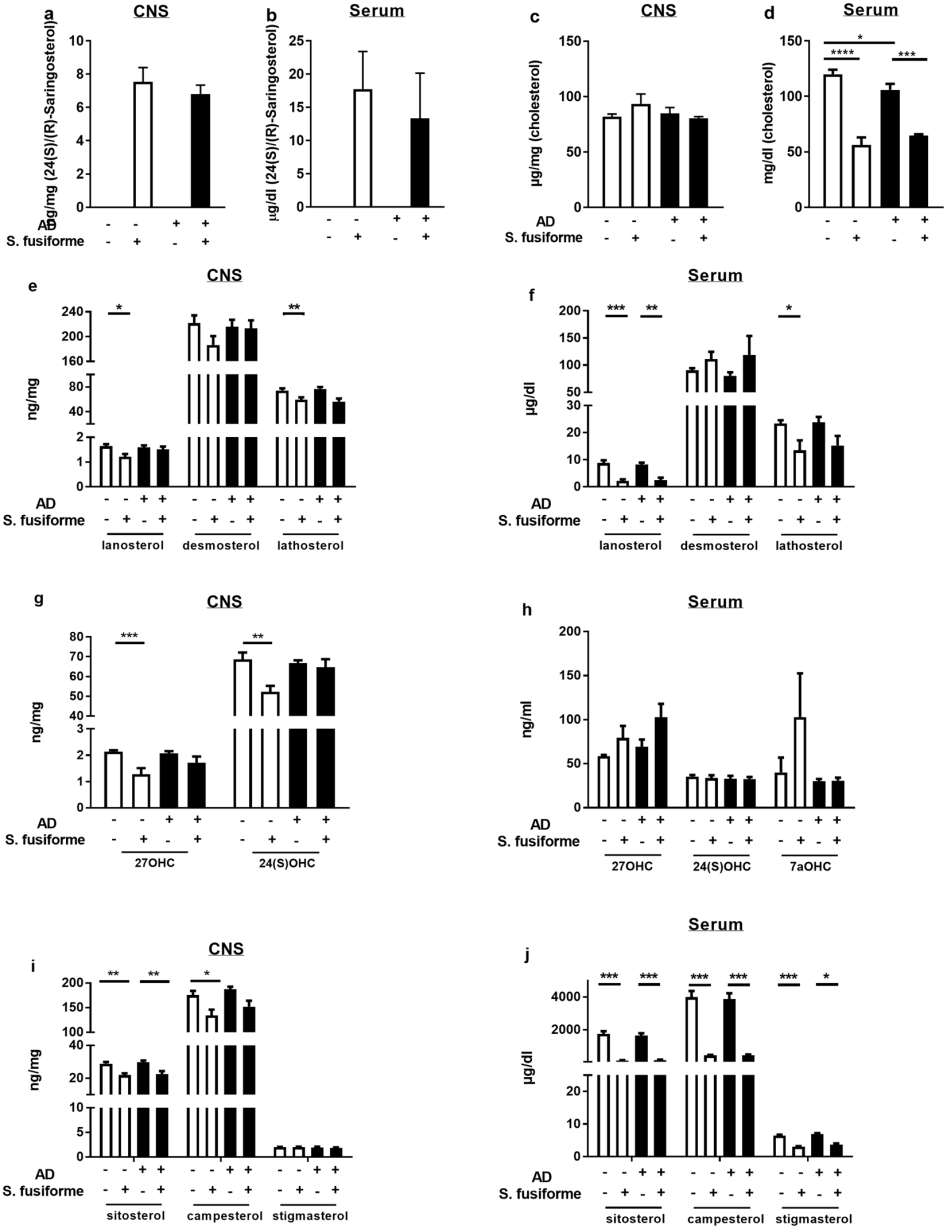
Dietary supplementation with *Sargassum fusiforme* reduces serum cholesterol, and increases 24(S)/(R)-Saringosterol in serum and cerebellum. Concentrations of 24(S)/(R)-Saringosterol (**a**,**b**), cholesterol (**c**,**d**), cholesterol precursors (**e**,**f**), cholesterol metabolites (**g**,**h**), and phytosterols (**i**,**j**) were determined in serum and cerebellum samples of WT and APPswePS1ΔE9 mice (AD) fed either control chow or chow supplemented with *Sargassum fusiforme*. 24(S)/(R)-Saringosterol concentration was affected by diet in CNS (F(1, 31) = 438.8, p < 0.0001) and serum (F(1, 31) = 30.05, p < 0.0001) as determined by ANOVA, and post-hoc Sidak’s test showed that this effect existed in both genotypes (WT CNS: p < 0.0001; WT serum: p = 0.0005; AD CNS: p < 0.0001; AD serum: p = 0.0157). Cholesterol concentration in serum was affected by diet (F(3, 31) = 80.59, p < 0.0001) for both genotypes (WT: p < 0.001; AD: p = 0.0002). Regarding the cholesterol precursors, concentration of lanosterol was affected by diet in CNS (F(1, 32) = 6.463, p = 0.0161) and serum (F(1, 31) = 38.28, p < 0.0001) of WT animals (CNS: p = 0.0265; serum: p = 0.0002) and AD animals (serum: p = 0.0024). Concentration of lathosterol was affected by diet in CNS (F(1, 32) = 19.32, p = 0.0001) and serum (F(1, 31) = 13.42, p = 0.0009) of WT animals (CNS: p = 0.0076; serum: p = 0.0474). Regarding the cholesterol metabolites, concentration of 27-hydroxycholesterol (27OHC) was affected by diet in CNS (F(1, 32) = 20.04, p = 0.0009) of WT animals (p = 0.0004). Concentration of 24(S)-hydroxycholesterol (24(S)OHC) was affected by diet in CNS (F(1, 31) = 8.071, p = 0.0079) of WT animals (p = 0.0079). Regarding the plant sterols, concentration of sitosterol was affected by diet in CNS (F(1, 32) = 30.62, p < 0.0001) and serum (F(1, 31) = 96.88, p < 0.0001) of WT animals (CNS: p = 0.0026; serum: p < 0.0001) and AD animals (CNS: p = 0.0028; serum: p < 0.0001). Concentration of campesterol was affected by diet in CNS (F(1, 32) = 17.02, p = 0.0002) and serum (F(1, 31) = 76.4, p < 0.0001) of WT animals (CNS: p = 0.0175; serum: p < 0.0001) and AD animals (serum: p < 0.0001). Concentration of stigmasterol was affected by diet in serum (F(1, 31) = 99.89, p < 0.0001) of WT animals (p < 0.0001) and AD animals (p < 0.0001). Bars represent mean ± SEM (n = 5). Abbreviations:7aOHC 7a-hydroxycholesterol.

To assess *in vivo* effects of *Sargassum fusiform*e APPswePS1ΔE9 mice were used as a model of AD^47^. These mice begin to develop Aβ plaques at 4 months of age and cognitive decline occurs from 6 months onwards^48,49^. From the age of 5 months APPswePS1ΔE9 mice and WT littermates were fed either standard chow or chow supplemented with 50% (w/w) dried crude *Sargassum fusiforme*. Ten weeks of *Sargassum fusiforme* dietary supplementation resulted in LXR activation in the CNS, evidenced by a cerebral induction of LXR response genes (*Abcg1, Scd1*) (Fig. 2a,b). *ApoE*, *Srebp-1c*, and *Abcg1* expression was not altered in animals treated with *Sargassum fusiforme* (Fig. 2c–e). Interestingly, 24(S)/(R)-Saringosterol was detectable in serum and in the cerebellum of animals that were fed *Sargassum fusiforme*, but not in those fed normal chow (Fig. 3a,b). As *Sargassum fusiforme* contains predominantly 24(S)-Saringosterol^34^, we postulate that this isoforms is the most abundant isoform present in animals fed *Sargassum fusiforme*. Animals fed a diet supplemented with *Sargassum fusiforme* did not show differences in total cholesterol content in the cerebellum (Fig. 3c), but showed a marked reduction in circulating cholesterol levels compared to chow fed animals (Fig. 3d). Levels of cholesterol precursors, cholesterol metabolites, and phytosterols were decreased in serum and cerebellum of animals supplemented with *Sargassum fusiforme* (Fig. 3e–j). On a standard chow diet, WT mice displayed significantly higher serum cholesterol levels than AD mice (Fig. 3d), as described previously^10,50^.

### *Sargassum fusiforme* improves memory and reduced Aβ plaque load in AD mice

**Figure 4:**
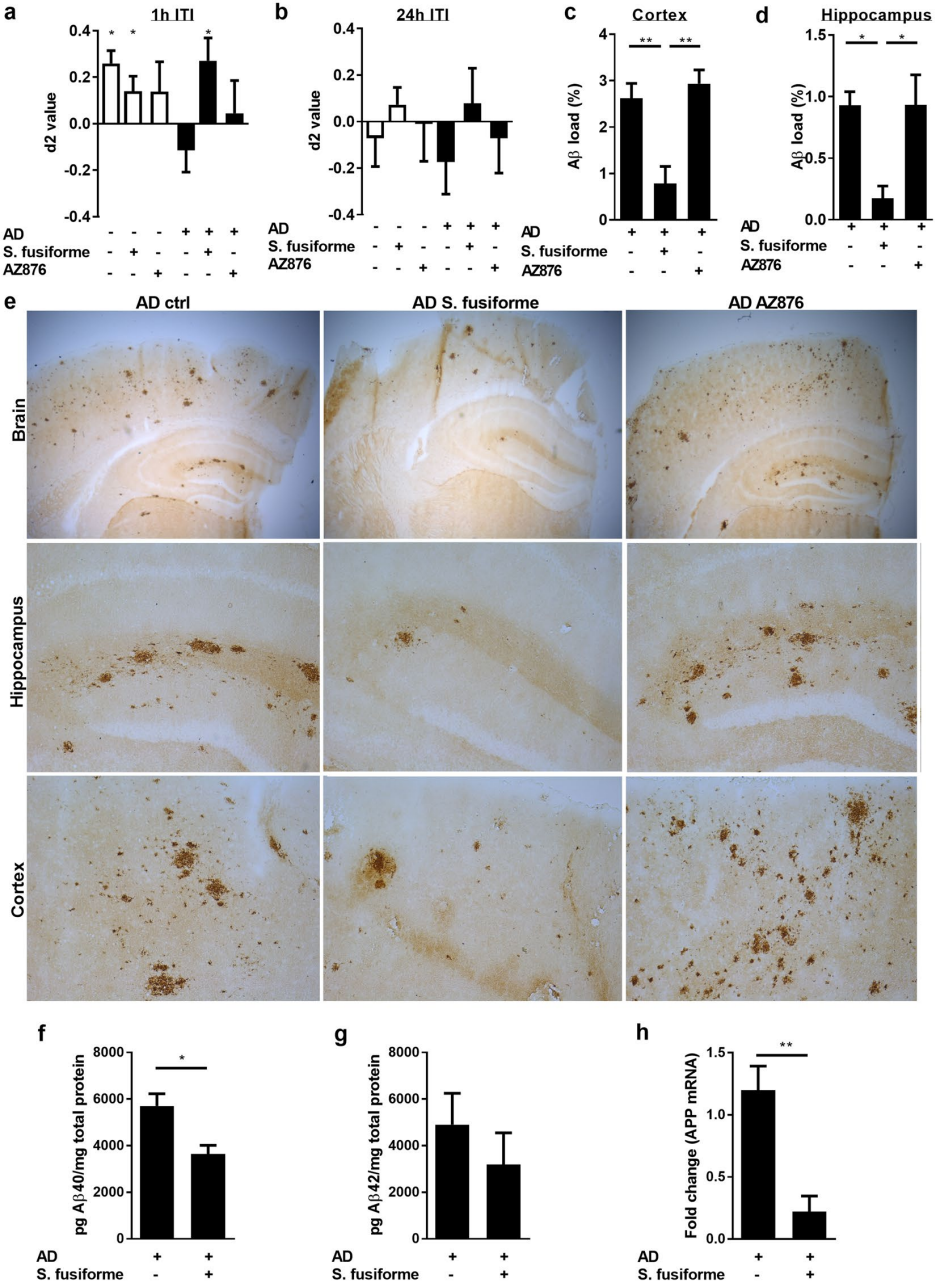
Dietary supplementation with *Sargassum fusiforme* reduces cognitive decline and Aβ plaque load in APPswePS1ΔE9 mice. (**a**,**b**) Cognitive functioning was determined using the object recognition task. The interval between first and second trial was set at 1 hour (1 h ITI) or 24 hours (24 h ITI). D2 value is calculated as the ratio between exploration time spent at the new object and the familiar object in the second trial, with d2 value > 0 indicating intact object memory. At 1 h ITI the object memory was found to be intact in WT animals on control diet (t(9) = 4.71, p = 0.0011, one sample t-test), in WT animals on *Sargassum fusiforme*-supplemented diet (t(9) = 2.166, p = 0.0585, one sample t-test), and in AD animals on *Sargassum fusiforme*-supplemented diet (t(8) = 2.77, p = 0.0243, one sample t-test). Bars represent mean ± SEM from two independent experiments (n ≥ 9 per treatment). (**c**–**e**) Aβ plaque load was quantified in cortex (**d**) and hippocampus (**e**) of APPswePS1ΔE9 mice using immunohistochemistry (n ≥ 5 per treatment). Aβ load is calculated as percentage of surface coverage, and was found to be decreased in AD animals fed *Sargassum fusiforme-*enriched chow in cortex (F (3, 24) = 25.79, p < 0.0001, ANOVA; Tukey’s post-hoc for diet effect in AD genotype: p = 0.0005) and hippocampus (F (3, 24) = 32.13, p < 0.0001, ANOVA; Tukey’s post-hoc for diet effect in AD genotype: p < 0.0001). Representative IHC staining of all groups is shown (**e**). (**f**,**g**) *Sargassum fusiforme* treated APPswePS1ΔE9 mice show a significant decrease in Aβ_40_ ((f); U = 6, n_ctrl_ = 11, n_extract_ = 5, p = 0.0133, Mann-Whitney) but not Aβ_42_ levels (**g**). (**h**) *Sargassum fusiforme* treated APPswePS1ΔE9 mice show a significant decrease in the mRNA expression of APP (U = 0, n_ctrl_ = 8, n_extract_ = 4, p = 0.0040, Mann-Whitney).

Next, we investigated the impact of dietary supplementation with crude *Sargassum fusiforme* on cognition and neuropathology in APPswePS1ΔE9 mice. To assess the influence of selective LXRα activation on cognition, animals were fed with the synthetic selective LXRα agonist AZ876. The open field test revealed no effect of genotype or diet on general locomotor activity, and anxiety (Table 1). Dietary supplementation with *Sargassum fusiforme* significantly improved object memory in APPswePS1ΔE9 mice in the object recognition task (ORT) at a 1 h but not a 24 h inter-trial interval (Fig. 4a,b). In contrast, selective LXRα activation did not impact object memory at either the 1 h or 24 h inter-trial interval. *Sargassum fusiforme*, but not AZ786, markedly decreased Aβ plaque load in the cortex (70% decrease; Fig. 4c,e) and hippocampus (81% decrease; Fig. 4d,e) in APPswePS1ΔE9 mice. In concordance, *Sargassum fusiforme* supplementation reduced Aβ40 protein and APP mRNA expression in the CNS of AD animals (Fig. 4f,h). *Sargassum fusiforme* did not significantly reduce insoluble Aβ42 (Fig. 4g). Reduced Aβ plaque load was not associated with an increased expression of phagocytic receptors such as Axl and MerTK (Fig. S4a,b)^51^. Yet, the reduced Aβ plaque load was associated with a reduced TREM2 expression, reflecting the expelled need for further Aβ clearance (Fig. S4c).

**Table 1:** Genotype and diet do not impact general locomotor activity or anxiety levels.

Genotype	Diet	TIZ centre (s)	TIZ walls and corners (s)	DM (m)	N
WT	Control	29.5 ± 7.7	570 ± 7.7	36.1 ± 1.6	12
AD	Control	29.4 ± 4.2	569.6 ± 4.3	39.0 ± 1.9	13
WT	*S. fusiforme*	30.9 ± 3.4	564.9 ± 4.3	42.0 ± 1.9	9
AD	*S. fusiforme*	33.5 ± 4.1	564.9 ± 5.2	47.6 ± 2.0	7
WT	AZ876	28.3 ± 4.3	569.2 ± 4.9	37.3 ± 2.4	11
AD	AZ876	31.7 ± 6.4	567.2 ± 6.4	39.9 ± 2.7	13

Results of open field test. Values are displayed as mean ± SEM. Abbreviations: TIZ, time in zone; DM, distance moved.

**Figure 5:**
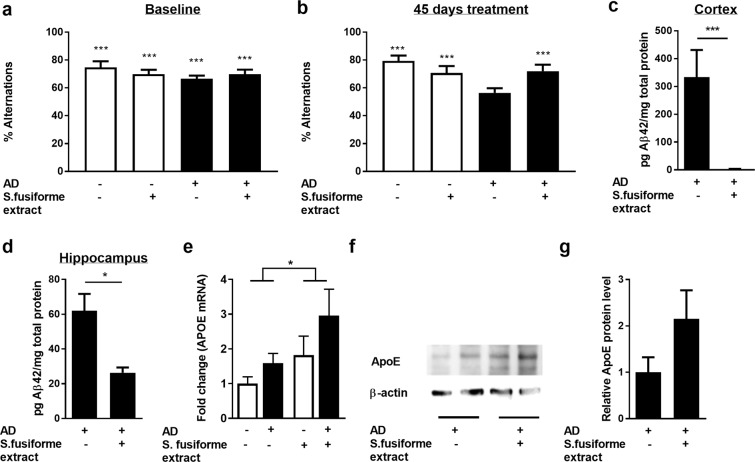
Dietary supplementation with a lipid extract of *Sargassum fusiforme* reduces cognitive decline and Aβ plaque load in APPswePS1ΔE9 mice. (**a**,**b**) A *Sargassum fusiforme* lipid extract was administered by gavage to determine the impact of *Sargassum fusiforme*-derived lipids on working memory in the spatial alteration Y maze. All mice showed an intact working memory at baseline ((a), spatial alteration > 50% (chance level); WT control p = 0.0002; WT *Sargassum fusiforme* p = 0.0008; AD control p < 0.0001, AD *Sargassum fusiforme* p = 0.0006, one-sample t-test). Upon six weeks of daily treatment (**b**), the untreated AD group displayed an impaired working memory (p = 0.085) while wild type mice and *Sargassum fusiforme* extract-treated mice retained their working memory (WT control p < 0.0001, WT *Sargassum fusiforme* p = 0.0018, AD *Sargassum fusiforme* p = 0.0008, one-sample t-test). (**c**,**d**) The extract-treated APPswePS1ΔE9 mice show a significant decrease in formic acid-extracted insoluble Aβ_42_ levels in the cortex ((c); U = 0, n_ctrl_ = 8, n_extract_ = 8, p = 0.0045, Mann-Whitney) and in the hippocampus ((d); U = 3; n_ctrl_ = 7, n_extract_ = 5, p = 0.0177, Mann-Whitney). (**e**–**g**) Mice treated with a lipid extract of *Sargassum fusiforme* show an increased mRNA (**e**) expression of *ApoE* in their CNS (F (1, 18) = 4.885, p = 0.0403, two-way ANOVA), although the increase in protein expression (**f**,**g**) does not reach significance. Data are shown as mean ± SEM.

To determine whether the lipid moiety of *Sargassum fusiforme* was sufficient to modulate the AD-related pathology, a purified lipid extract of *Sargassum fusiforme* was administered daily by oral gavage to APPswePS1ΔE9 and WT littermates for 45 days. Working memory was significantly improved in APPswePS1ΔE9 mice, resulting in the prevention of the spatial working deficit (Fig. 5a,b). In line with these findings, a significant reduction in insoluble Aβ_42_ was observed in the cortex (99% decrease; Fig. 5c) and hippocampus (57% decrease; Fig. 5d) of the treated APPswePS1ΔE9 mice. Notably, treatment with the *Sargassum fusiforme* lipid extract resulted an increased expression of ApoE in the CNS (Fig. 5e–g), which indicates active LXR signaling.

### *Sargassum fusiforme* feeding does not lead to liver steatosis

**Figure 6:**
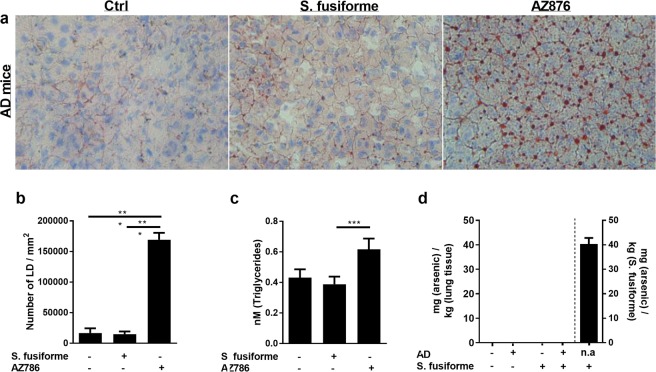
*Sargassum fusiforme* supplementation does not result in liver steatosis, elevated circulating triglyceride levels, or arsenic accumulation in the lungs. (**a**) An ORO staining was performed to define lipid droplets in liver samples of APPswePS1ΔE9 mice fed normal chow or *Sargassum fusiforme*/AZ876-enriched chow. (**b**) Quantification of the ORO staining shown in (1) calculated as number of lipid droplets per mm^2^ of liver tissue. (**c**) AZ876 but not *Sargassum fusiforme* increases the circulating levels of triglycerides in and APPswePS1ΔE9 mice (F (2, 31) = 4.056, p = 0.0272, ANOVA; Tukey’s post-hoc for diet effect: p = 0.0305). (**d**) Arsenic was assesed in dried *Sargassum fusiforme* and lung samples of WT and APPswePS1ΔE9 mice (AD) fed normal chow or chow supplemented with *Sargassum fusiforme*.

While synthetic LXR agonists are well-known to induce liver steatosis in mice^52^*, Sargassum fusiforme* supplementation did not induce liver steatosis in APPswePS1ΔE9 mice, as evidenced by the absence of lipid droplets within the liver of these animals (Fig. 6a,b). Supplementation with AZ876 resulted in a marked increase in lipid droplets in the liver (Fig. 6a,b). Likewise, while AZ876 increased circulating triglyceride levels, *Sargassum fusiforme* did not impact the level of triglycerides (Fig. 6c). Similar findings were observed in WT mice fed chow supplemented with *Sargassum fusiforme* or AZ876 (data not shown). *Sargassum fusiforme* is known to contain relatively high levels of (in)organic arsenic that can accumulate in the body^53^. Although relatively high levels of arsenic were measured in dried *Sargassum fusiforme* (40.4 mg/kg), no arsenic accumulation was detectable in lung tissue of the animals that consumed *Sargassum fusiforme* (Fig. 6d).

### 24(S)-Saringosterol increases microglial Aβ clearance and reduces neuronal Aβ release

**Figure 7:**
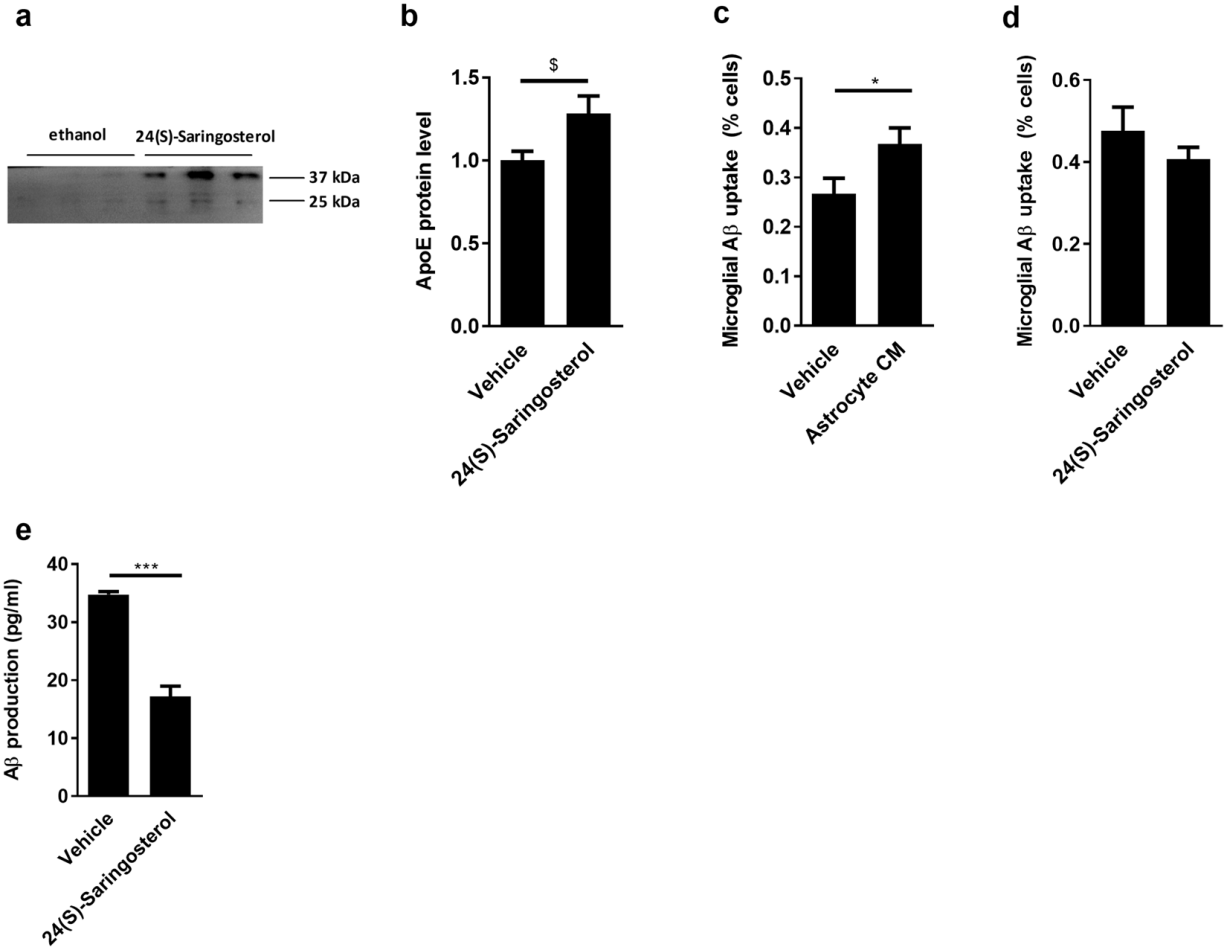
24(S)-Saringosterol increases astrocytic ApoE production and microglial Aβ clearance and reduces neuronal Aβ release *in vitro*. (**a**,**b**) Primary astrocytes were treated with 10 µM 24(S)-Saringosterol for 2 consecutive days and medium was collected 48 h after the last treatment. Western Blot was used to measure secreted ApoE in the cellular supernatant. 24(S)-Saringosterol increased ApoE secretion (t(5) = 2.531, p = 0.0524, unpaired t-test). Representative WB bands are displayed in the figure. (**c**) Primary murine microglia were examined for their capacity to internalize FAM-labeled Aβ_1−42_. Microglia were incubated for 1 h with fluorescently-labeled FAM-Aβ_1−42_ and 40% conditioned medium derived from astrocytes treated with vehicle (ethanol) or 24(S)-Saringosterol (10 µM). Data are depicted as the percentage of cells that phagocytosed FAM-Aβ_1−42_. 24(S)-Saringosterol increased microglial uptake of FAM-Aβ_1−42_ (t(14) = 2.218, p = 0.0436, unpaired t-test). (**d**) To define a direct effect on microglial uptake of Aβ_1−42,_ primary murine microglia were exposed to 24(S)-Saringosterol prior to defining FAM-labeled Aβ_1−42_ uptake. (**e**) N2aAPPswe cells were stimulated with vehicle (ethanol) or 24(S)-Saringosterol (10 µM). ELISA was used to define the release of Aβ42. 24(S)-Saringosterol decreased Aβ42 production (t(5) = 8.126, p = 0.0005, unpaired t-test).

To obtain insight into the mechanisms by which *Sargassum fusiforme*-derived 24(S)-Saringosterol impacts AD pathogenesis we used well-established *in vitro* models that mimic AD-related pathological processes. Supplementation of astrocytes with 24(S)-Saringosterol resulted in an increased ApoE secretion (Fig. 7a,b). Furthermore, supplementation of microglia with conditioned medium of 24(S)-Saringosterol-treated astrocytes promoted microglial clearance of Aβ_1−42_ (Fig. 7c). 24(S)-Saringosterol did not directly impact the capacity of microglia to internalize Aβ_1−42._ (Fig. 7d). Finally, 24(S)-Saringosterol was found to reduce the release of Aβ_42_ using neuronal N2a cells overexpressing APP (Fig. 7e).

## Discussion

Our data show that dietary supplementation with the brown seaweed *Sargassum fusiforme* improves cognitive function and reduces Aβ load in APPswePS1ΔE9 mice without inducing liver steatosis. Our data further suggest the involvement of the stereospecific oxyphytosterol 24(S)-Saringosterol, a selective LXRβ activator, in the observed effects on cognition and Aβ plaque load. Considering that selective LXRα activation did not improve cognitive decline or Aβ plaque deposition in APPswePS1ΔE9 mice, our findings suggest that natural and possibly also synthetic LXRβ agonists are attractive options for the treatment of neurodegenerative disorders such as AD.

Phytosterols, which can cross the blood-brain barrier, have been reported to activate LXRs without inducing hypertriglyceridemia and hepatic steatosis, rendering them potentially suitable candidates for treatment of neurodegenerative disorders^11^. Yet, in contrast with reported data on LXR activation by phytosterols common in a Western diet^34–39^, we observed very little if any effect of stigmasterol, brassicasterol, β-sitosterol, fucosterol, or a commercial mix of phytosterols, on activation of LXRα and LXRβ *in vitro*. The discrepancy between our data and the literature may be explained by differences in the experimental design, such as phytosterol concentration, incubation time, and cell type and assay used to measure LXR activation. For instance, we used a cell-based luciferase reporter assay and CNS-derived cell lines, as well as HEK293.T and COS-7 cells, to define LXR activation and cellular specificity in LXR activation, while other studies used cell-free assays^38^. Furthermore, we used phytosterol concentrations in a physiological range that can be reached through dietary supplementation (1–25 µM), whereas other studies used concentrations far exceeding this physiological range up to 200 µM^36^. Of note, compared to synthetic LXR agonists, the reported ability of phytosterols commonly present in a Western diet to activate LXRs is relatively limited^34–39^. In line with this, no alterations were observed in the expression of LXR-response genes in the brain of ABCG5-knockout mice despite high cerebral levels of common phytosterols^26^. In summary, our data indicate that phytosterols present in a Western type diet do not activate LXRs at concentrations that can be reached through dietary supplementation^11^.

Out of the tested extracts of Asian plants, the extract of *Sargassum fusiforme* most potently activated LXRβ *in vitro*. This is in line with the recently reported LXRβ-activating effects of *Sargassum fusiforme*-derived 24(S)-Saringosterol^34^. In *Sargassum fusiforme*–fed animals, 24(S)-Saringosterol was detected in the cerebellum in concentrations equal to common oxysterols, suggesting a good CNS bioavailability. The amount of 24(S)-Saringosterol detected in the CNS was comparable to previously reported concentrations of other dietary phytosterols in the CNS^26,32,33^. With respect to these findings, we found that cerebral expression of LXR-response genes was upregulated in animals fed *Sargassum fusiforme*-enriched chow, further supporting the ability of *Sargassum fusiforme* to activate LXRβ. The route of entry of 24(S)-Saringosterol to the CNS remains unclear. Further research is warranted to define the role of ApoE and SR-B1 in facilitating the transport of 24(S)-Saringosterol into the CNS^11,32^. Moreover, since 24(S)-Saringosterol is oxydated plant sterol, we hypothesize that 24(S)-Saringosterol can enter the CNS via transcellular diffusion, comparable to other oxidized cholesterol derivatives such as 24(S)-Hydroxycholesterol^54^. Interestingly, in contrast to 24(S)-Saringosterol, all plant sterol concentrations decreased in serum and CNS upon feeding *Sargassum fusiforme*. It can be hypothesized that 24(S)-Saringosterol activates intestinal and hepatic Abcg5/g8 transporters and thereby drives the enterohepatic secretion of plant sterols, thereby lowering circulating levels of sterols^55^. The latter hypothesis is in line with the strong reduction in circulating cholesterol levels we found in this study.

Our data indicate that the LXRα selective agonist AZ876 does not significantly counteract cognitive decline or reduce Aβ plaque load in APPswePS1ΔE9 mice. However, no active LXR signaling was found in the CNS of AZ876 treated animals, which suggest poor CNS bioavailability of AZ876 and consequently does not allow us to conclude that LXRα activation does not impact AD pathology. On the other hand, while both LXRα and LXRβ are expressed in the murine CNS, expression of LXRβ is 15-fold higher compared to LXRα^56,57^. Hence, the relative contribution of LXRα activation upon full-LXR agonist treatment is therefore expected to be limited. The latter studies may suggest that the beneficial impact of pan-LXR agonists on cognitive decline in AD models likely relies on LXRβ activation.

Synthetic LXR agonists that reduce AD pathology and improve cognitive performance induce hypertriglyceridemia and liver steatosis^2,13,14,24,25,27–30^. *Sargassum fusiforme* supplementation counteracted cognitive decline of APPswePS1ΔE9 transgenic mice without inducing hepatic steatosis. This may be explained by selective hepatic LXRβ activation, since animals fed AZ786 (2 mg/kg body weight) displayed a marked increase of liver lipid droplets, indicative of liver steatosis. Alternatively, the presence of other protective compounds present in *Sargassum fusiforme* such as fucosterol may prevent hepatic steatosis^11^. This is supported by the observation that activation of LXRβ by synthetic agonists was also reported to induce plasma and hepatic hypertriglyceridemia^58^.

We show that dietary supplementation with crude or lipid extract of *Sargassum fusiforme* reduces Aβ load in AD mice. Our *in vitro* data indicate that factors released by 24(S)-Saringosterol-treated astrocytes promote microglial clearance of Aβ1–42. Within the brain, astrocytes are the major source of the LXR response gene *Apoe*. ApoE is well-known to increase microglial clearance of Aβ^59^, possibly via inhibiting cellular uptake of Aβ^60^. Of interest, 24(S)-Saringosterol and a lipid extract of *Sargassum fusiforme* increased ApoE protein expression in astrocytes and the CNS respectively, which suggests a role for ApoE in the observed increase in microglial Aβ clearance. In addition, we provide evidence that 24(S)-Saringosterol decreases the release of Aβ by neurons *in vitro*. Together, these findings indicate that 24(S)-Saringosterol impacts both the clearance and generation of Aβ_42_. These findings are of particular interest, as beneficial effects of LXR agonists on cognition are not necessarily accompanied by a reduced Aβ plaque load^14,41^. Recently, it has been reported that other constituents of *Sargassum fusiforme*, such as fucosterol, fucoidan, and fucoxantin improve learning and memory deficiencies in pharmacological models for cognitive impairment^61–63^. Although synergism among constituents of *Sargassum fusiforme* is likely, future studies should determine whether 24(S)-Saringosterol is sufficient to improve cognitive performance and AD pathology.

Although relatively high levels of arsenic were measured in extracts of *Sargassum fusiforme*, no arsenic was detectable in lung tissue, which generally represents a major reservoir for this element^64^. Arsenic present in *Sargassum fusiforme* may therefore be rapidly excreted, degraded or absorbed in low amounts in the intestine.

In summary, our data indicate that phytosterols present in a Western type diet do not activate LXRs at concentrations that can be reached through dietary supplementation. However, a crude lipid extract of the edible seaweed *Sargassum fusiforme* did selectively activate LXRβ *in vitro*. Here, we show for the first time that dietary supplementation with *Sargassum fusiforme* significantly improves memory performance and reduces Aβ plaque load in a well-established AD model. The accumulation of the natural LXRβ agonist 24(S)-Saringosterol in the CNS suggests its involvement in the neuroprotective impact of *Sargassum fusiforme*. Collectively, our findings point to 24(S)-Saringosterol-containing *Sargassum fusiforme* as being a novel candidate for dietary supplementation to prevent or modulate neurodegenerative disorders such as Alzheimer’s disease.

## Materials and Methods

### Preparation of plant extracts

Indigenous Asian plants (*Asparagus racemosus*, *Azadirachta indica*, *Cassia fistula*, *Curcuma aromatica*, *Datura metel*, *Piper retrofractum*, *Sargassum fusiforme*, *Senna tora*, and *Terminalia chebula*), were selected based on their use in Asian traditional medicine as cognition enhancers. Extracts from all plants, with exception of *Sargassum fusiforme*, were prepared by the maceration method. All plants were dried in a hot air oven at 50 °C. The dried samples were finely powdered and soaked in 95% ethanol overnight at room temperature and filtered with Whatman filter paper No. 3 (Sigma-Aldrich, Bornem, Belgium). Three consecutive filtrates were pooled and evaporated in a vacuum rotary evaporator at 40 °C. The crude lipid fraction of *Sargassum fusiforme* was extracted using an adaptation of the Folch method^65^. Briefly, *Sargassum fusiforme* was harvested by hand in early spring, washed in sea water, boiled for three hours and dried in a hot air oven. Next, dried *Sargassum fusiforme* (Clearspring Ltd, London, UK) was soaked overnight at room temperature in a 2:1 (v/v) chloroform and methanol (both VWR, Leuven, Belgium) mixture. The chloroform/methanol extract was evaporated under a N_2_ stream and the remaining lipid fraction was dissolved in ethanol. Using gas chromatography/mass spectrometry, 24(S)-Saringosterol content of dried *Sargassum fusiforme* was determined to be 69.4 ng/mg.

### Cell culture

Immortalized human embryonic kidney cells (HEK293.T), human microglia (CHME3; a kind gift from prof. dr. M. Tardieu, Universite Paris-Sud, France^66^), human oligodendrocytes (MO3.13), mouse neuroblastoma expressing APPswe (N2a/APPswe; a kind gift from prof. dr. T.W. Kim, Colombia University, USA^67^), and monkey kidney cells (COS7) were used for *in vitro* experiments. All cell lines were cultured in DMEM (Sigma-Aldrich) containing 10% heat-inactivated FCS (Invitrogen, Merelbeke, Belgium) and 100 U penicillin/100 µg streptomycin/ml (Invitrogen), at 37 °C/5% CO_2_. For phytosterol treatment, cells were incubated for 18 hours in culture medium without FCS containing the Eastern plants extracts, brassicasterol (Sigma-Aldrich), β-sitosterol, (Sigma-Aldrich), fucosterol (Sigma-Aldrich), stigmasterol (analytic confirmed purity of 99,9%), phytosterol mix (containing 60% β-sitosterol, 25% campesterol, and 15% stigmasterol; kindly provided by Ingmar Wester Raisio, Finland), T0901317 (Cayman Chemicals, Huissen, the Netherlands), ethanol (VWR), or DMSO (Sigma-Aldrich).

### Luciferase-based nuclear receptor reporter assay

To determine the ability of plant extracts and phytosterols to bind LXRα and LXRβ, a luciferase-based reporter assay was performed using the ONE-Glo^TM^ Luciferase Assay System kit (Promega, Leiden, the Netherlands), according to manufacturer’s instructions. Cell lines were transfected with bacterial plasmid constructs expressing luciferase under control of the promotor region of the ligand-binding domain for LXRα or LXRβ^68^. Cells were grown to 50–60% confluency in 60 mm plates, transfected with 1.8 μg of plasmid DNA including 0.2 μg pGAL4hLXRα or pGAL4hLXRβ, 1 μg pG5-TK-GL3, and 0.6 μg of pCMV-β-galactosidase, using JetPEI (Polyplus-transfection SA, Illkirch, France) as transfection reagent. Following treatment, cells were lysed in lysis buffer (25 mM Glycyl-Glycine, 15 mM MgSO_4_, 4 mM EGTA, and 1x Triton; all from Sigma). To correct for transfection efficacy, β-galactosidase activity was measured using lysate diluted 1:10 in B-gal buffer, consisting of 20% 2-Nitrophenyl β-D-galactopyranoside (ONGP; Sigma) and 80% (v/v) Buffer Z (0.1 M Na_2_HPO_4_, 10 mM KCl, 1 mM MgSO_4,_ and 3.4 µl/ml β-mercaptoethanol; all from Sigma). Luminescence and absorbance (410 nM) was measured using the FLUOstar Optima (BMG Labtech, Ortenberg, Germany).

### Animals and diet

Male APPswePS1ΔE9 mice (Radboud University Medical Center, Nijmegen^47^) were backcrossed with female C57Bl6/J mice (Harlan Netherlands B.V., Horst, The Netherlands) to obtain male APPswePS1ΔE9 and wild-type (WT) littermates. Animals were housed in a conventional animal facility at Hasselt University. Two series of animal experiments were conducted, the first using dietary supplementation of dried *Sargassum fusiforme*, and the second using gavage of a lipid extract of *Sargassum fusiforme*. A timeline of the animal experiments is depicted in Supplemental Fig. 1.

For the first series, experimental diets consisted of either powdered standard chow (Teklad 2018; Harlan Netherlands B.V.) or chow supplemented with 50% (w/w) pulverized dried *Sargassum fusiforme* (Clearspring Ltd, London, UK) or the synthetic LXRα agonist AZ876 (2 mg/kg body weight, kindly provided by AstraZeneca, Mölndal, Sweden^69^). The amount of 24(S)-Saringosterol used was 69.4 ng/mg dried *Sargassum fusiforme*. Based on an estimated intake of 7 g dry food/day, the amount of daily 24(S)-Saringosterol corresponds to 242 µg/day. Animals were divided into three groups and received either the *Sargassum fusiforme* containing diet (APPswePS1ΔE9 n = 13; C57Bl6/J n = 10), AZ876 containing diet (APPswePS1ΔE9 n = 14; C57Bl6/J n = 13), or the control diet (APPswePS1ΔE9 n = 13; C57Bl6/J n = 12) from 5 until 7.5 months of age.

The second batch of animals was divided into two groups and received either the *Sargassum fusiforme*-derived lipid extract (APPswePS1ΔE9 n = 9; C57Bl6/J n = 8), or the vehicle (APPswePS1ΔE9 n = 11; C57Bl6/J n = 12) from 6 until 7.5 months of age. Lipids of *Sargassum fusiforme* were extracted using the Folch-method^65^. Briefly, dried *Sargassum fusiforme* was pulverized and soaked in a 2:1 (v/v) chloroform:methanol (VWR, Leuven, Belgium) mixture for 16 hours at room temperature. Next, the mixture was sonicated (10 min), filtered, and subsequently evaporated by heating the extract until 60 °C. The remaining extract was washed in ethanol, sonicated (10 min), and evaporated. Finally, the extract was dissolved in corn oil (Vita D’or, the Netherlands) as vehicle and sonicated prior to *in vivo* supplementation. Using gas chromatography/mass spectrometry, 24(S)/(R)-Saringosterol content was measured to be 332 mg/dl (7,744 mM). The extract was administered by daily gavage (200 µl/25 g body weight) for a period of 45 days, corresponding to a daily lipid intake of 3.5 g seaweed/day (664 µg 24(S)/(R)-Saringosterol/day). In a previous study we defined that *Sargassum fusiforme* contains predominantly 24(S)-Saringosterol^34^. Hence, we expect that this isoforms is the most abundant isoform present in our *in vivo* experiments.

One week prior to the start of the behavioral experiments all animals were housed individually. Animals were kept at an inverse 12 h light/12 h dark cycle, and behavioral experiments were performed during the dark phase of the cycle. Cognitive performance was scored blind. All animal procedures were performed in accordance with institutional guidelines and approved by the ethical committee for animal experiments of Hasselt University.

### Behavioral tasks

The object recognition task (ORT) was conducted after 9 weeks of *Sargassum fusiforme* supplementation when the mice were ~8 months old, as described previously^70,71^. In brief, all animals were habituated to the arena over 2 days, in one trial of 4 min per day. In each habituation trial, two different objects were placed symmetrically in the center of the arena, about 10 cm from the wall. Four different objects were available and at the end of the habituation period each animal had encountered all four objects once. After one resting day, the experiment started. During the first trial (T1) two identical objects (samples) were placed in the arena. After exploring the samples for 4 min, the animal was placed back in its home cage. Subsequently, after a predetermined delay interval (1 or 24 hours), the second trial of 4 minutes (T2) was performed using one familiar object from trial 1 and one new object. Exploration time for each object during T1 and T2 was recorded manually using a personal computer. Sitting on the object or biting in the object was not considered exploratory behavior. Discrimination index d2 in T2 (d2 = [(exploration time for the novel object) − (exploration time for the familiar object)]/(total exploration time in T2)) was calculated as measure for object memory.

The Y-maze spontaneous alternation test^72^ was conducted at baseline and after 6 weeks of extract treatment. The maze used for the Y-maze spatial alteration task consisted of three arms of grey Perspec of equal length (40 × 10 × 15 cm) (labeled A, B and C), separated at an angle of 120°. The animals were free to explore the maze for 6 minutes. Afterwards, the working memory is calculated in order of percentage of alternations defined as: (number of triads/(total number of entries −2)) * 100. The number of triads was recorded manually and was defined as subsequently entering the three different arms, while entering an arm was defined as placing both hind paws in that arm. After each trial, the maze was cleaned with 70% ethanol to prevent olfactory cues.

The open field test (OF) was conducted as described previously^73^, after 9.5 weeks of *Sargassum fusiforme* treatment. In short, animals were placed in a square, Perspex box (50 cm × 50 cm × 30 cm), with an open top, grey walls, and a white floor. Video tracking with a computerized system (Etho Vision^TM^, Noldus, Wageningen, The Netherlands) was used to record and analyse movements and position of the animals, in order to determine time in zone (TIZ) and distance moved (DM) over the 10-minute trial^14^.

### Tissue sample preparation

Mice were anaesthetized with Nembutal (100 mg/kg i.p., CEVA Logistics, Brussels, Belgium) prior to transcardial perfusion with Ringer’s solution. Blood was collected and centrifuged for 10 min at 200 g to obtain serum, which was snap-frozen in liquid nitrogen. Liver samples were divided into two parts and either directly snap-frozen or frozen in Tissue-Tek O.C.T embedding compound (Sakura Finetek, Berchem, Belgium). Brains were divided into three parts prior to snap-freezing; the cerebellum and two hemispheres. The right hemisphere was preserved for immunohistochemistry and frozen in Tissue-Tek O.C.T. embedding compound. The rostral half of the left hemisphere was preserved for mRNA expression analyses with quantitative PCR. The cerebellum was preserved for sterol analyses.

### Aβ quantification

The right hemisphere of the crude *Sargassum fusiforme*-treated mice was cut in the coronal plane with a Leica CM1900UV cryostat (Leica Microsystems, Wetzlar, Germany) to obtain 10 μm sections. Sections were mounted on glass slides, air-dried overnight, and stored at room temperature until used. After blocking of endogenous peroxidases by incubation for 30 min with 3% H_2_O_2_ in methanol, the sections were incubated for 30 min with blocking solution (5% bovine serum albumin in 1x TBS) followed by overnight 4 °C primary antibody incubation in blocking solution (clone 3D6, Amyloid-β Antibody 1:8000). The sections were then incubated in biotinylated goat anti-human IgG (BA-3000, 1:1000, Vector Laboratories, Burlingame, CA), and the immunoreactivity was developed by the ABC avidin–biotin peroxidase KIT (PK-6100, Vector) for 1 hour, and 5 min with diaminobenzidine (DAB, ImmPACT SK-4105, Vector). Between every other step, the slides were rinsed (3×) with TBS/0.3% Triton-X 100. The sections were counterstained with haematoxylin for 30 seconds and coverslipped. Digital images of the sections were obtained using a Leica DM 2000 LED microscope (Leica Microsystems, Diegem, Belgium) equipped with hardware and software from Leica Application suite software (Leica Microsystems). The Aβ plaque load was quantified in 3 sections per brain in 5 animals per group using Fiji ImageJ, by defining the pixel intensity of Aβ plaques in the total cortical or hippocampal area at Bregma −1.5 to −2.5.

Next, left hippocampus and cortex were dissected from the brains of the *Sargassum fusiforme* extract-treated APPswePS1ΔE9 mice and homogenated in a 2% SDS TBS-T buffer. Aβ_42_ levels were quantified and related to the protein content in the starting homogenate using an Aβ_42_ ELISA (Invitrogen, USA), according to the manufacturer’s instructions^74^. In brief, the homogenates were sonicated twice and mixture centrifuged to generate an SDS-insoluble fraction (21000 g, 10 min). The SDS-insoluble pellet was sonicated twice in 70% formic acid (FA), yielding the FA-extracted insoluble Aβ_42_ fraction.

Mouse neuroblastoma (N2a) cells, stably overexpressing APP, were cultured in high glucose DMEM medium supplemented with 10%FCS and 100 U pen/100 µg strep/ml (37 °C, 5% CO2) Next, the cells were incubated for 24 h with ethanol (vehicle control) or 10 µM 24(S)-Saringosterol, purified from *Sargassum fusiforme* as described earlier^34^. Aβ_42_ levels were detected in supernatant using an Aβ_42_ ELISA (Invitrogen, USA), according to the manufacturer’s instructions.

### Determination of astrocytic ApoE secretion

To generate 24(S)-Saringosterol-astrocyte conditioned medium, mouse primary astrocytes were seeded in a 24-well plate with a density of 250000 cells/well. Next, astrocytes were treated with either 10 µM purified 24(S)-Saringosterol or ethanol for 2 consecutive days after which medium was replenished and collected 2 days afterwards. Next, ApoE secretion was determined using western blot. Equal amounts of cellular supernatant (40 µl) were loaded and separated via electrophoresis through a 10% sodium dodecyl sulfate polyacrylamide gel (SDS-PAGE). Afterwards, proteins were transferred onto a PVDF membrane (VWR, Belgium), and blots were blocked (5% non-fat dry milk TBS-Tween solution) for 1 h at room temperature. Next, membranes were incubated with rabbit anti-ApoE primary antibody (ABBIOTEC, USA). After washing the membranes with TBS-Tween, the blots were incubated with swine anti-rabbit HRP conjugated antibody (WAKO, Japan). Finally, membranes were washed in TBS and protein bands were detected using Pierce^TM^ ECL Plus Western Blotting Substrated and ImageQuant^TM^ LAS 4000 mini. ImageJ (http://imagej.nih.gov/ij/) was used to quantify the protein bands.

### Aβ phagocytosis assay

Primary murine cells were isolated and cultured as described previously^75^. In short, mixed glial cultures were prepared from postnatal d0 mouse cerebral cortices of C57Bl6/J mice (Harlan Netherlands B.V.) and cultured in high glucose DMEM medium supplemented with 10% FCS and 100 U pen/100 µg strep/ml. Mixed glial cultures were used to generate microglia-enriched glial cultures by separating the microglia from the astrocyte monolayer using orbital shaking followed by purification via differential adhesion to plastic after 14 days in culture. Purified microglia were seeded on poly-L-lysine (5 µg/ml; Sigma-Aldrich) coated 96-well plates with a density of 50000 cells/well. Primary microglia were cultured in high glucose DMEM medium supplemented with 10% FCS, 100 U pen/100 µg strep/ml and 15% L929 conditioned medium.

The capacity of primary microglia to phagocytose FAM-labeled Aβ_1−42_ (Eurogentec, Seraing Belgium) was assessed with a plate-based assay. Twenty hours after seeding the microglia, cells were treated with 24(S)-Saringosterol-astrocyte conditioned medium (40%) and FAM-labeled Aβ_1−42_ was added for 1 h at a final concentration of 500 nM. Next, medium was removed and 100 µl 0.2% trypan blue in PBS (pH 4.4) was added to quench extracellular Aβ. After aspiration, fluorescence was measured at 485/535 nm excitation/emission. Finally, to normalize for cell number, 100 µl DAPI in PBS was added, incubated for 10 min and medium was aspirated. Fluorescence was subsequently measured in a plate reader using 360 nm/465 nm excitation/emission wavelengths.

### Sterol profile and triglyceride content determination

Sterol profiles were determined in serum and cerebellum. Preceding the analysis, samples were spun in a speed vacuum dryer (Savant AES 1000) at 12 mbar for 24 h to relate individual sterol concentrations to their dry weight. The sterols were extracted from the dried tissue by placing them in a mixture of chloroform:methanol (2:1) for 24 h at 4 °C. Sterol levels were determined by gas chromatography/mass spectrometry (GC/MS) as described previously^32,76^. Triglyceride contents of serum were determined using the GOD-PAP method with enzymatic reagent kits according to manufacturer’s instructions (DiaSys Diagnostic Systems, Holzheim, Germany).

### Determination of arsenic concentration

Arsenic halogenide concentrations in lung tissues were analyzed using inductively coupled plasma-optical emission spectrometry (ICP–OES, Agilent Technologies, 700 Series, Belgium). Tissues were oven-dried and digested with 70–71% HNO_3_ in a heat block. Prior to analysis, lung samples and dried *Sargassum fusiforme* were diluted 1:10 in 1% nitric acid. Concentrations were measured using a standard calibration curve (NIST Spinach 1570a).

### RNA isolation and RT-Q-PCR

Brain tissue was homogenized in Qiazol (Qiagen, Venlo, The Netherlands) preceding RNA isolation, and total RNA was prepared using the RNeasy mini kit (Qiagen), according to manufacturer’s instructions. RNA concentration and quality was determined with a NanoDrop spectrophotometer (Isogen Life Science, IJsselstein, The Netherlands). RNA was reverse transcribed to cDNA using the qScript^TM^ cDNA synthesis kit (Quanta Biosciences, Gaithersburg, USA). As previously described^77,78^, quantitative PCR was subsequently conducted on a StepOnePlus detection system (Applied biosystems, Gaasbeek, Belgium). Relative quantification of gene expression was performed using the comparative Ct method. Data were normalized to the most stable reference genes (Cyca and Hmbs). Primers were chosen according to literature or designed using Primer-Express (http://www.ncbi.nlm.nih.gov/tools/primer-blast), and details of primers used are shown in Table 2.

**Table 2:** Quantitative PCR primers.

Gene symbol	Gene name	Forward and reverse primer
*Cyca*	Cyclin-A	F: TGGGATTGTACCACAGCTCCA R: CTCATGATGACTGCAGCAAACC
*Hmbs*	Hydroxymethylbilane synthase	F: GATGAAGCCATTGCTGAACTTG R: GTCTCCTTGGGTATCCGATGTC
*Abca1*	ATP-binding cassette, sub-family A, member 1	F: CCCAGAGCAAAAAGCGACTC R: GGTCATCATCACTTTGGTCCTTG
*Abcg1*	ATP-binding cassette, sub-family G, member 1	F: CAAGACCCTTTTGAAAGGGATCT R: GCCAGAATATTCATGAGTGTGGAC
*ApoE*	Apolipoprotein E	F: CCTGAACCGCTTCTGGGATT R: GCTCTTCCTGGACCTGGTCA
*App*	Amyloid precursor protein	F: CGA ACC CTA CGA AGA AGC CAC R: GCT TTC TGG AAA TGG GCA TGT TC
*Axl*	Axl receptor tyrosine kinase	F: GGA ACC CAG GGA ATA TCA CAG G R: AGT TCT AGG ATC TGT CCA TCT CG
*Mertk*	Proto-oncogene tyrosine-protein kinase Mer	F: TGC GTT TAA TCA CAC CAT TGG A R: TGC CCC GAG CAA TTC CTT TC
*Scd1*	Stearoyl-CoA desaturase 1	F: TGCGATACACTCTGGTGCTCA R: CTCAGAAGCCCAAAGCTCAGC
*Srebp-1c*	Sterol regulatory element binding protein 1c	F: GGAGCCATGGATTGCACATT R: GCTTCCAGAGAGGAGCCCAG
*Trem2*	Triggering receptor expressed on myeloid cells 2	F: CTG GAA CCG TCA CCA TCA CTC R: CGA AAC TCG ATG ACT CCT CGG

Nucleotide sequence of primers used for quantitative PCR. F denotes forward primer, R denotes reverse primer.

### Experimental design and statistical analyses

All statistical analyses were performed using GraphPad Prism 7^TM^ and are reported as mean ± SEM. D’Agostino and Pearson omnibus normality test was used to test normal distribution. Unless specified differently, one-way ANOVA (post-hoc: Tukey) or two-tailed unpaired Student t-test was used for normally distributed data sets. The Kruskal-Wallis (Dunns post hoc comparison) or Mann–Whitney analysis was used for data sets assessed not to be normally distributed. The ORT discrimination index d2 (compared to the 0) and spatial alteration Y-maze performance (compared to the 50% chance level) was analyzed using the one-sample t-test. Animals that did not reach the minimum of 4 s exploration in T1 or T2 were excluded from further analysis^71,79^. Data from RT-Q-PCR was analyzed using two-way ANOVA with treatment and genotype as independent variables. In all data sets, extreme values were excluded by means of Dixon’s principles of exclusion of extreme values^80,81^. Significance levels are denoted as follows: ^$^p < 0.10 *p < 0.05, **p < 0.01, ***p < 0.001 or ****p < 0.0001.

### Ethical approval

All animal procedures were performed in accordance with institutional guidelines and approved by the ethical committee for animal experiments of Hasselt University.

## Supplementary information

Supplementary figures

## Data Availability

The datasets used and/or analyzed during the current study are available from the corresponding author on reasonable request.

## References

[1] Parihar MS, Hemnani T (2004). Alzheimer’s disease pathogenesis and therapeutic interventions. Journal of clinical neuroscience: official journal of the Neurosurgical Society of Australasia.

[2] Jansen D (2012). Cholesterol and synaptic compensatory mechanisms in Alzheimer’s disease mice brain during aging. Journal of Alzheimer’s disease: JAD.

[3] Jones L (2010). Genetic evidence implicates the immune system and cholesterol metabolism in the aetiology of Alzheimer’s disease. PloS one.

[4] Kolsch H (2010). Alterations of cholesterol precursor levels in Alzheimer’s disease. Biochim Biophys Acta.

[5] Kolsch H (2004). Altered levels of plasma 24S- and 27-hydroxycholesterol in demented patients. Neurosci Lett.

[6] Mulder M (2009). Sterols in the central nervous system. Current opinion in clinical nutrition and metabolic care.

[7] Popp J (2012). Cholesterol metabolism is associated with soluble amyloid precursor protein production in Alzheimer’s disease. J Neurochem.

[8] Shobab LA, Hsiung GY, Feldman HH (2005). Cholesterol in Alzheimer’s disease. The Lancet. Neurology.

[9] Stefani M, Liguri G (2009). Cholesterol in Alzheimer’s disease: unresolved questions. Current Alzheimer research.

[10] Vanmierlo T (2010). Alterations in brain cholesterol metabolism in the APPSLxPS1mut mouse, a model for Alzheimer’s disease. Journal of Alzheimer’s disease: JAD.

[11] Vanmierlo, T. *et al*. Plant sterols: friend or foe in CNS disorders? *Progress in Lipid Research*, 10.1016/j.plipres.2015.01.003 (2015). 10.1016/j.plipres.2015.01.003 25623279

[12] Fassbender K (2001). Simvastatin strongly reduces levels of Alzheimer’s disease beta -amyloid peptides Abeta 42 and Abeta 40 *in vitro* and *in vivo*. Proc Natl Acad Sci USA.

[13] Jiang Q (2008). ApoE Promotes the Proteolytic Degradation of Aβ. Neuron.

[14] Vanmierlo T (2011). Liver X receptor activation restores memory in aged AD mice without reducing amyloid. Neurobiology of aging.

[15] Baez-Becerra C (2018). Receptor Agonist GW3965 Regulates Synaptic Function upon Amyloid Beta Exposure in Hippocampal Neurons. Neurotoxicity research.

[16] Lei C (2017). Amelioration of amyloid beta-induced retinal inflammatory responses by a LXR agonist TO901317 is associated with inhibition of the NF-kappaB signaling and NLRP3 inflammasome. Neuroscience.

[17] Stachel SJ (2016). Identification and *in Vivo* Evaluation of Liver X Receptor beta-Selective Agonists for the Potential Treatment of Alzheimer’s Disease. Journal of medicinal chemistry.

[18] Sandoval-Hernandez AG (2016). Liver X Receptor Agonist Modifies the DNA Methylation Profile of Synapse and Neurogenesis-Related Genes in the Triple Transgenic Mouse Model of Alzheimer’s Disease. Journal of molecular neuroscience: MN.

[19] Zelcer N, Tontonoz P (2006). Liver X receptors as integrators of metabolic and inflammatory signaling. The Journal of clinical investigation.

[20] Bensinger SJ, Tontonoz P (2008). Integration of metabolism and inflammation by lipid-activated nuclear receptors. Nature.

[21] Hong C, Tontonoz P (2014). Liver X receptors in lipid metabolism: opportunities for drug discovery. Nature reviews. Drug discovery.

[22] Nelissen K (2012). Liver X receptors regulate cholesterol homeostasis in oligodendrocytes. Journal of neuroscience research.

[23] Sodhi RK, Singh N (2013). Liver X receptors: Emerging therapeutic targets for Alzheimer’s disease. Pharmacological Research.

[24] Zelcer N (2007). Attenuation of neuroinflammation and Alzheimer’s disease pathology by liver x receptors. Proc Natl Acad Sci USA.

[25] Riddell DR (2007). The LXR agonist TO901317 selectively lowers hippocampal Aβ42 and improves memory in the Tg2576 mouse model of Alzheimer’s disease. Molecular and Cellular Neuroscience.

[26] Vanmierlo T (2011). Cerebral accumulation of dietary derivable plant sterols does not interfere with memory and anxiety related behavior in Abcg5-/- mice. Plant Foods Hum Nutr.

[27] Grefhorst A (2002). Stimulation of lipogenesis by pharmacological activation of the liver X receptor leads to production of large, triglyceride-rich very low density lipoprotein particles. J Biol Chem.

[28] Kim GH (2015). Hepatic TRAP80 selectively regulates lipogenic activity of liver X receptor. The Journal of clinical investigation.

[29] Repa JJ (2000). Regulation of mouse sterol regulatory element-binding protein-1c gene (SREBP-1c) by oxysterol receptors, LXRalpha and LXRbeta. Genes Dev.

[30] Schultz JR (2000). Role of LXRs in control of lipogenesis. Genes & Development.

[31] Fricke CB (2007). Increased plant sterol and stanol levels in brain of Watanabe rabbits fed rapeseed oil derived plant sterol or stanol esters. The British journal of nutrition.

[32] Jansen PJ (2006). Dietary plant sterols accumulate in the brain. Biochimica et Biophysica Acta (BBA) - Molecular and Cell Biology of Lipids.

[33] Vanmierlo T (2012). Dietary intake of plant sterols stably increases plant sterol levels in the murine brain. J Lipid Res.

[34] Chen Z (2014). 24(S)-Saringosterol from edible marine seaweed Sargassum fusiforme is a novel selective LXRbeta agonist. J Agric Food Chem.

[35] El Kharrassi Y (2014). Biological activities of Schottenol and Spinasterol, two natural phytosterols present in argan oil and in cactus pear seed oil, on murine miroglial BV2 cells. Biochem Biophys Res Commun.

[36] Hoang MH (2012). Fucosterol is a selective liver X receptor modulator that regulates the expression of key genes in cholesterol homeostasis in macrophages, hepatocytes, and intestinal cells. Journal of agricultural and food chemistry.

[37] Kaneko E (2003). Induction of intestinal ATP-binding cassette transporters by a phytosterol-derived liver X receptor agonist. J Biol Chem.

[38] Plat J, Nichols JA, Mensink RP (2005). Plant sterols and stanols: effects on mixed micellar composition and LXR (target gene) activation. Journal of Lipid Research.

[39] Yang C (2004). Disruption of cholesterol homeostasis by plant sterols. The Journal of clinical investigation.

[40] Burg VK (2013). Plant sterols the better cholesterol in Alzheimer’s disease? A mechanistical study. J Neurosci.

[41] Koivisto H (2014). Special lipid-based diets alleviate cognitive deficits in the APPswe/PS1dE9 transgenic mouse model of Alzheimer’s disease independent of brain amyloid deposition. J Nutr Biochem.

[42] Shi C (2011). beta-sitosterol inhibits high cholesterol-induced platelet beta-amyloid release. J Bioenerg Biomembr.

[43] McDaniel AL (2013). Phytosterol feeding causes toxicity in ABCG5/G8 knockout mice. The American journal of pathology.

[44] Plat J (2014). Protective role of plant sterol and stanol esters in liver inflammation: insights from mice and humans. PloS one.

[45] Cheng Z (2012). Interaction of ergosterol with bovine serum albumin and human serum albumin by spectroscopic analysis. Molecular biology reports.

[46] Sudhamalla B, Gokara M, Ahalawat N, Amooru DG, Subramanyam R (2010). Molecular dynamics simulation and binding studies of beta-sitosterol with human serum albumin and its biological relevance. *The journal of physical chemistry*. B.

[47] Jankowsky JL (2001). Co-expression of multiple transgenes in mouse CNS: a comparison of strategies. Biomolecular Engineering.

[48] Garcia-Alloza M (2006). Characterization of amyloid deposition in the APPswe/PS1dE9 mouse model of Alzheimer disease. Neurobiology of Disease.

[49] Minkeviciene R (2008). Age-related decrease in stimulated glutamate release and vesicular glutamate transporters in APP/PS1 transgenic and wild-type mice. Journal of Neurochemistry.

[50] Hooijmans CR (2009). DHA and cholesterol containing diets influence Alzheimer-like pathology, cognition and cerebral vasculature in APPswe/PS1dE9 mice. Neurobiology of disease.

[51] Calabro P, Gragnano F, Pirro M (2017). Cognitive Function in a Randomized Trial of Evolocumab. The New England journal of medicine.

[52] Mashek DG, Khan SA, Sathyanarayan A, Ploeger JM, Franklin MP (2015). Hepatic lipid droplet biology: Getting to the root of fatty liver. Hepatology (Baltimore, Md.).

[53] Rose M (2007). Arsenic in seaweed—Forms, concentration and dietary exposure. Food and Chemical Toxicology.

[54] Bjorkhem I (1987). Correlation between serum levels of some cholesterol precursors and activity of HMG-CoA reductase in human liver. Journal of lipid research.

[55] Jones PJH (2018). Progress and perspectives in plant sterol and plant stanol research. Nutr Rev.

[56] Annicotte JS, Schoonjans K, Auwerx J (2004). Expression of the liver X receptor alpha and beta in embryonic and adult mice. Anat Rec A Discov Mol Cell Evol Biol.

[57] Zhang Y (2014). An RNA-sequencing transcriptome and splicing database of glia, neurons, and vascular cells of the cerebral cortex. The Journal of neuroscience: the official journal of the Society for Neuroscience.

[58] Kirchgessner TG (2016). Beneficial and Adverse Effects of an LXR Agonist on Human Lipid and Lipoprotein Metabolism and Circulating Neutrophils. Cell Metab.

[59] Ries M, Sastre M (2016). Mechanisms of Abeta Clearance and Degradation by Glial. Cells. Frontiers in aging neuroscience.

[60] Fu Y (2016). Apolipoprotein E lipoprotein particles inhibit amyloid-beta uptake through cell surface heparan sulphate proteoglycan. Molecular neurodegeneration.

[61] Alghazwi, M., Smid, S., Musgrave, I. & Zhang, W. *In vitro* studies of the neuroprotective activities of astaxanthin and fucoxanthin against amyloid beta (Abeta1-42) toxicity and aggregation. *Neurochem Int*, 10.1016/j.neuint.2019.01.010 (2019). 10.1016/j.neuint.2019.01.010 30639263

[62] Oh, J. H., Choi, J. S. & Nam, T. J. Fucosterol from an Edible Brown Alga Ecklonia stolonifera Prevents Soluble Amyloid Beta-Induced Cognitive Dysfunction in Aging Rats. *Mar Drugs* **16**, 10.3390/md16100368 (2018). 10.3390/md16100368 PMC6213915 30301140

[63] Hu P (2016). Structural elucidation and protective role of a polysaccharide from Sargassum fusiforme on ameliorating learning and memory deficiencies in mice. Carbohydr Polym.

[64] Kenyon EM (2008). Tissue distribution and urinary excretion of inorganic arsenic and its methylated metabolites in C57BL6 mice following subchronic exposure to arsenate in drinking water. Toxicology and applied pharmacology.

[65] Folch J, Lees M, Sloane Stanley GH (1957). A simple method for the isolation and purification of total lipides from animal tissues. J Biol Chem.

[66] Cross AK, Woodroofe MN (1999). Chemokines induce migration and changes in actin polymerization in adult rat brain microglia and a human fetal microglial cell line *in vitro*. Journal of neuroscience research.

[67] Sun Y, Yao J, Kim TW, Tall AR (2003). Expression of liver X receptor target genes decreases cellular amyloid beta peptide secretion. J Biol Chem.

[68] Bories G (2013). Liver X receptor activation stimulates iron export in human alternative macrophages. Circulation research.

[69] van der Hoorn J (2011). Low dose of the liver X receptor agonist, AZ876, reduces atherosclerosis in APOE*3Leiden mice without affecting liver or plasma triglyceride levels. British journal of pharmacology.

[70] Rutten K (2005). The selective PDE5 inhibitor, sildenafil, improves object memory in Swiss mice and increases cGMP levels in hippocampal slices. Behavioural brain research.

[71] Sik A, van Nieuwehuyzen P, Prickaerts J, Blokland A (2003). Performance of different mouse strains in an object recognition task. Behavioural brain research.

[72] Ohno M (2004). BACE1 deficiency rescues memory deficits and cholinergic dysfunction in a mouse model of Alzheimer’s disease. Neuron.

[73] Mulder M (2004). Low-density lipoprotein receptor-knockout mice display impaired spatial memory associated with a decreased synaptic density in the hippocampus. Neurobiology of Disease.

[74] Steinerman JR (2008). Distinct pools of beta-amyloid in Alzheimer disease-affected brain: a clinicopathologic study. Archives of neurology.

[75] O’Meara, R. W., Ryan, S. D., Colognato, H. & Kothary, R. Derivation of enriched oligodendrocyte cultures and oligodendrocyte/neuron myelinating co-cultures from post-natal murine tissues. *Journal of visualized experiments: JoVE*, 10.3791/3324 (2011). 10.3791/3324 PMC3217647 21876528

[76] Lütjohann D (2002). Profile of cholesterol-related sterols in aged amyloid precursor protein transgenic mouse brain. Journal of Lipid Research.

[77] Bogie JF (2013). Myelin alters the inflammatory phenotype of macrophages by activating PPARs. Acta neuropathologica communications.

[78] Bogie JF (2012). Myelin-derived lipids modulate macrophage activity by liver X receptor activation. PLoS One.

[79] Akkerman S (2012). Object recognition testing: methodological considerations on exploration and discrimination measures. Behavioural brain research.

[80] Dixon WJ (1959). Ratios involving extreme values. Ann Math Stat.

[81] Dixon WJ (1959). Analysis of extreme values. Ann Math Stat.

