# Supplementary material for: Dietary Sargassum fusiforme improves memory and reduces amyloid plaque load in an Alzheimer’s disease mouse model
Source: Sci Rep. 2019 Mar 20;9:4908. doi: 10.1038/s41598-019-41399-4 (PMC6426980; doi:10.1038/s41598-019-41399-4)
Supplement: Supplementary file 1 — Supplementary figures [file 41598_2019_41399_MOESM1_ESM.pdf]

Dietary *Sargassum fusiforme* improves memory and reduces amyloid plaque load in an Alzheimer's disease mouse model

Jeroen Bogie[a]*, Cindy Hoeks[a]*, Melissa Schepers[a,i], Assia Tiane[a,i], Ann Cuypers[b], Frank Leijten[c], Yupyn Chintapakorn[d], Thiti Suttiyut[d], Surachai Pornpakakul[e], Dicky Struik[f], Anja Kerksiek[g], Hong-Bing Liu[h], Niels Hellings[a], Pilar Martinez-Martinez[i], Johan W. Jonker[f], Ilse Dewachter[a], Eric Sijbrands[c], Jochen Walter[j], Jerome Hendriks[a], Albert Groen[k], Bart Staels[l], Dieter Lütjohann[g], Tim Vanmierlo[a,i#] and Monique Mulder[c#]

Corresponding author: Tim Vanmierlo; tim.vanmierlo@uhasselt.be, T: +32 11 26 9228 Department of Immunology and Biochemistry, Biomedical research institute, Hasselt University, Martelarenlaan 42, 3500 Hasselt Belgium

[*,#] equal contribution

[a] Department of Immunology and Biochemistry, Biomedical research institute, Hasselt University, Martelarenlaan 42, 3500 Hasselt Belgium

[b] Centre for Environmental Sciences, Hasselt University, Martelarenlaan 42, 3500 Hasselt, Belgium

[c] Department of Internal Medicine, Laboratory of Vascular Medicine, Erasmus University Medical Center, Wytemaweg 80, 3015 CN, Rotterdam, the Netherlands

[d] Center of Excellence in Environment and Plant Physiology, Department of Botany, Faculty of Science, Chulalongkorn University, Bangkok 10330, Thailand

[e] Department of Chemistry, Faculty of Science, Chulalongkorn University, Bangkok 10330, Thailand

[f] Section of Molecular Metabolism and Nutrition, Department of Pediatrics, University of Groningen, University Medical Center Groningen, Hanzeplein 1, 9713 GZ Groningen, The Netherlands

[g] Institute for Clinical Chemistry and Clinical Pharmacology, Sigmund-Freud-Str. 25, D-53127 Bonn, Germany

[h] Key Laboratory of Marine Drugs, Ministry of Education, School of Medicine and Pharmacy, Ocean University of China, Yushan Road 5, 266003 Qingdao, China

[i] School for mental health and neuroscience, Maastricht University, Universiteitssingel 50 6229ER Maastricht, The Netherlands

[j] Department of Neurology, Molecular Cell Biology, University of Bonn, Sigmund-Freud-Str. 25, 53127 Bonn, Germany

[k] Department of Medical Biochemistry, Academic Medical Center, University of Amsterdam, Meibergdreef 9, 1105 AZ, Amsterdam, the Netherlands

[l] University of Lille - EGID, Inserm, U1011, University Hospital CHU, Institut Pasteur de Lille, F-59019 Lille, France

Supplementary figures

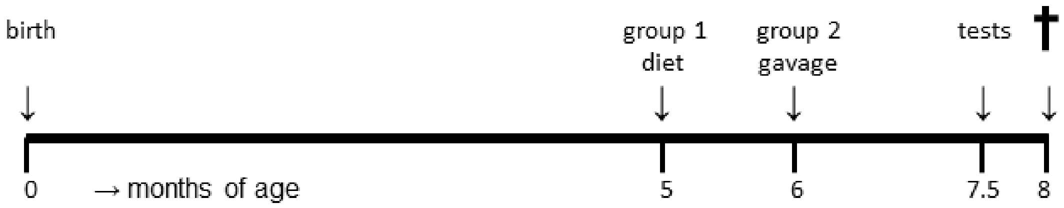

Supplementary Figure 1: Timeline of animal experiments. In this schematic overview the start of supplementation of *Sargassum fusiforme* is depicted, with group 1 started on dietary supplementation of the whole seaweed at five months of age, and group 2 started on daily gavage of a lipid extract of the seaweed at six months of age. For both groups, behavioral experiments (denoted as "tests" in the figure) were performed around 7.5 months of age, and animals were sacrificed at eight months of age. Details of *Sargassum fusiforme* supplementations and behavioral experiments are given in the Materials and Methods section.

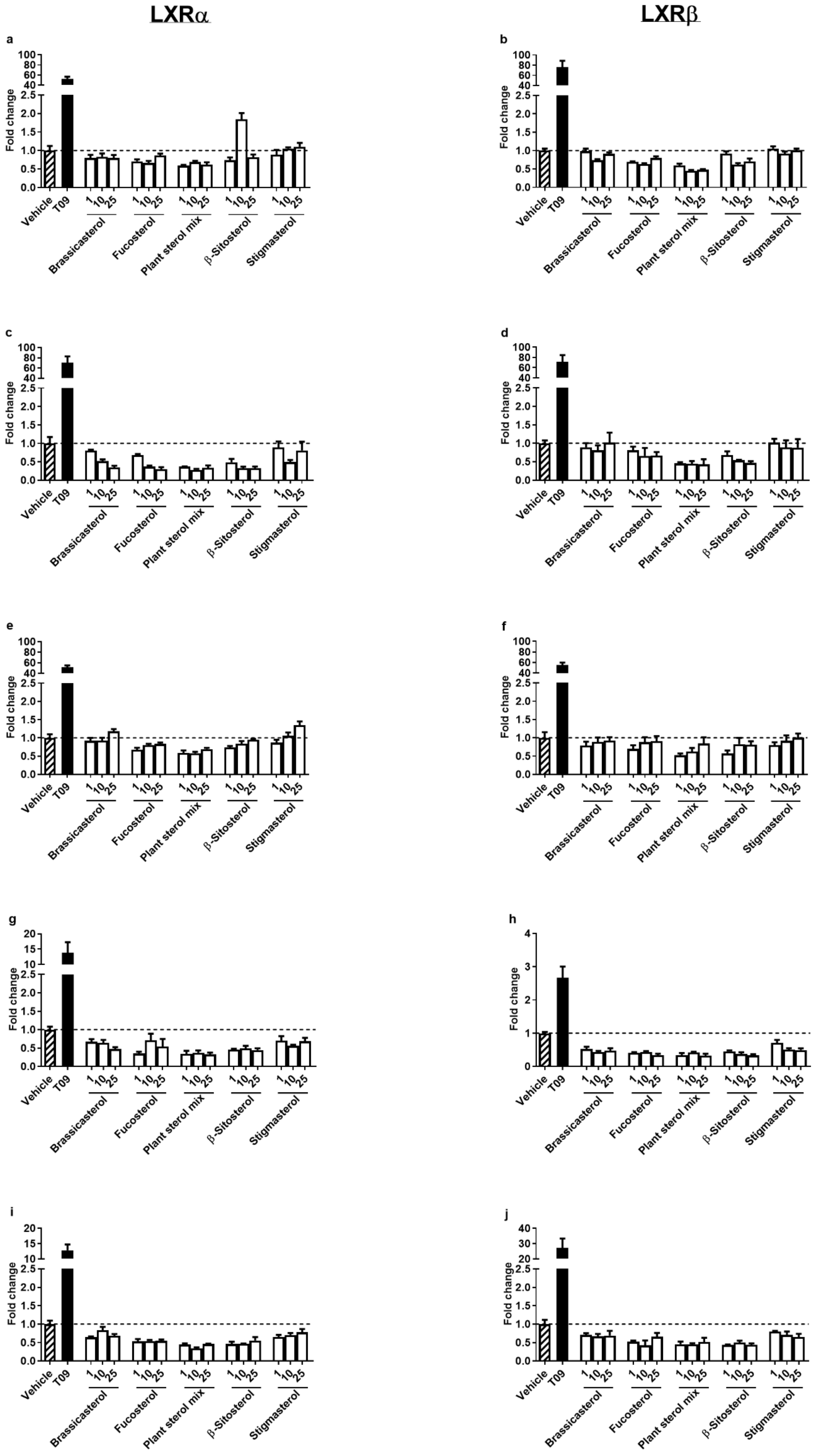

Supplementary figure 2, legend on next page

Supplementary figure 2: Phytosterols derived from a Western type diet hardly activate LXRα or LXRβ. LXR activation was assessed with a luciferase-based reporter assay. HEK293.T (a, b), COS7 (c, d), CHME3 (e, f), MO3.13 (g, h), and Na2/APPswe (i, j) cells were stimulated for 18 hours with vehicle (striped bar/dotted line), 1 µM T0901317 (black bar), or 1, 10 or 25 µM of the specified phytosterol prior to measuring luminescence. In all cell lines tested, a Kruskal-Wallis effect was found (HEK293.T: LXRα $\chi^2(16)=37.87$, $p=0.0016$, LXRβ $\chi^2(16)=44.84$, $p=0.0001$; COS7: LXRα $\chi^2(16)=41.76$, $p=0.0004$, LXRβ $\chi^2(16)=32.18$, $p=0.0095$; CHME3: LXRα $\chi^2(16)=43.89$, $p=0.0002$, LXRβ $\chi^2(16)=25.2$, $p=0.0664$; MO3.13: LXRα $\chi^2(16)=34.91$, $p=0.0041$, LXRβ $\chi^2(16)=34.85$, $p=0.0042$; Na2/APPswe: LXRα $\chi^2(16)=42.76$, $p=0.0003$, LXRβ $\chi^2(16)=34.58$, $p=0.0045$; all datasets analysed using Kruskal-Wallis test). All results are displayed as fold change compared to vehicle control. Bars represent mean ± SEM (n≥3).

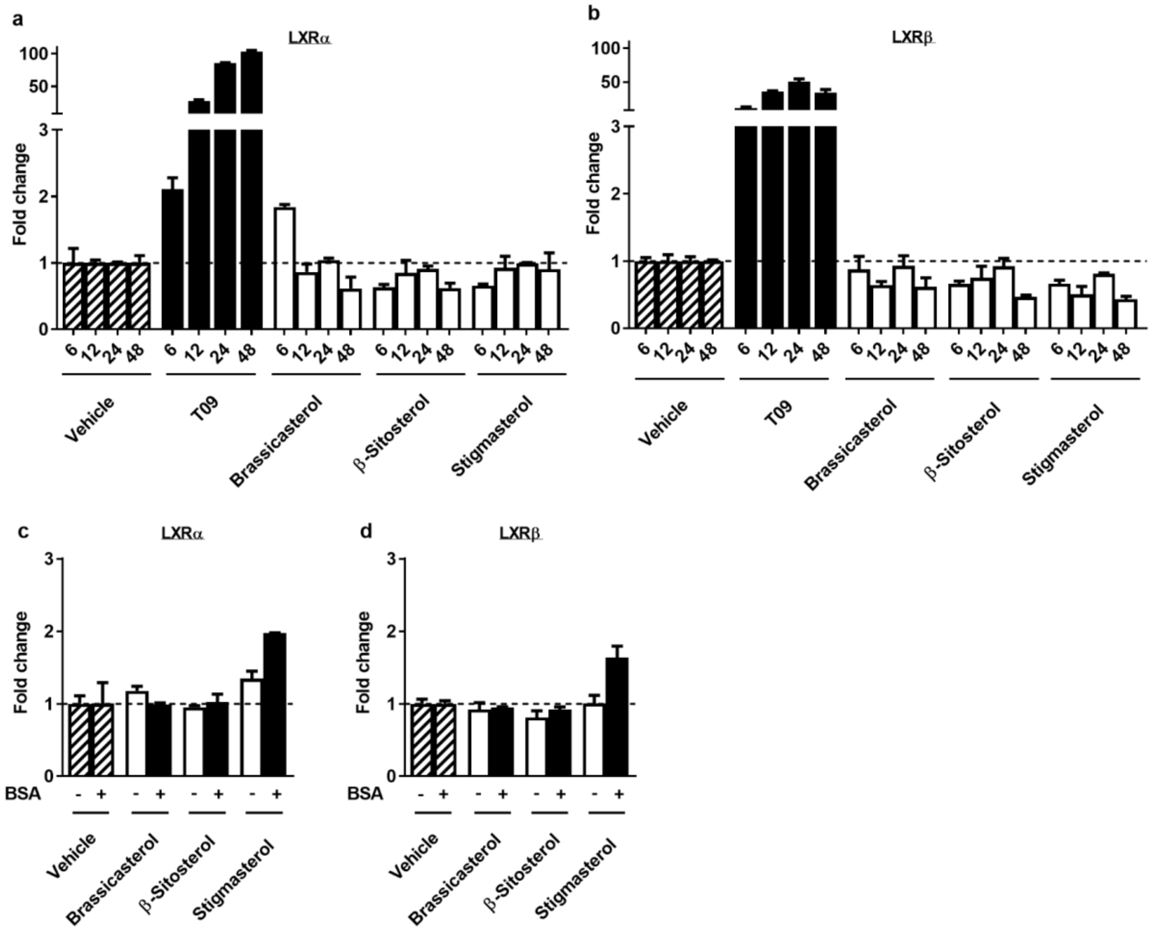

Supplementary figure 3: Prolonged incubation or complexation of common Western diet-containing phytosterols with BSA does not affect LXR activation. LXR activation was assessed with a luciferase-based reporter assay. (a, b) To determine if LXR activation of phytosterols increases in time, CHME3 cells were incubated for 6, 12, 24 or 48 hours with vehicle (striped bars/dotted line), 1μM T0901317 (black bars) or 25 μM of the specified phytosterol. Prolonged incubation with T0901317, but not phytosterols, increased LXR activation (LXRα $\chi^2(19)=31.58$, $p=0.0348$, LXRβ $\chi^2(19)=34.21$, $p=0.0174$; all datasets analysed using Kruskal-Wallis test). (c, d) BSA-enriched medium was used to determine if serum proteins facilitate LXR activation by phytosterols. No difference was found between groups (LXRα $\chi^2(7)=11.55$, $p=0.1165$, LXRβ $\chi^2(7)=7.689$, $p=0.3608$; all datasets analysed using Kruskal-Wallis test). All results are displayed as fold change compared to vehicle control (striped bars/dotted line). Bars represent mean ± SEM ($n\geq2$).

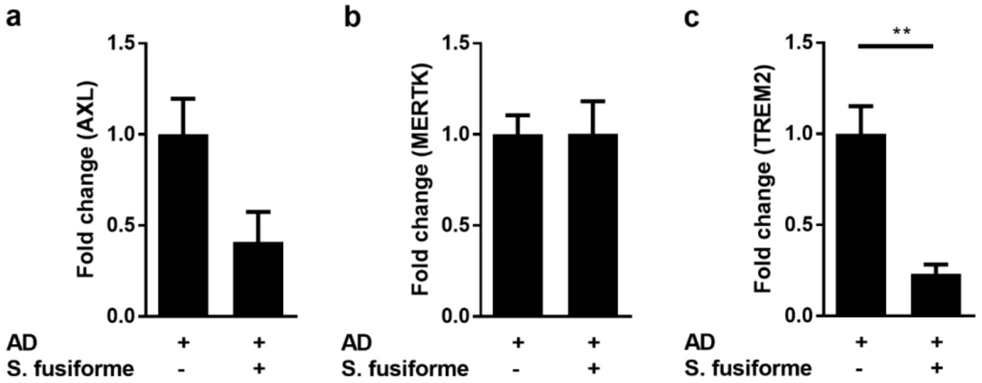

Supplementary figure 4: Dietary supplementation with *Sargassum fusiforme* decreases *Trem2* but not *Axl* and *Mertk*. Gene expression of *Axl* (a), *MerTK* (b), and *Trem2* (c) was measured in the brain of APPswePS1ΔE9 mice (AD) fed normal chow or chow supplemented with *Sargassum fusiforme*. *Trem2* expression was decreased in animals fed *Sargassum fusiforme* ((c); U=0; n_{ctrl} =8, $n_{extract}$=4, p=0.0040, Mann-Whitney). Gene expression was normalized to *Cyca* and *Hmbs*, and expressed as fold change compared to WT mice fed the control diet. Bars represent mean ± SEM (n≥4).
